# Changes in health-related quality of life scores among low-income patients on social welfare programs in Japan during the COVID-19 pandemic: a single-center repeated cross-sectional study

**DOI:** 10.1186/s12889-022-14597-5

**Published:** 2022-11-22

**Authors:** Satoshi Wakata, Daisuke Nishioka, Yukio Takaki

**Affiliations:** 1 Kamigyo Clinic, 482-2 Hanaguruma-Cho, Senbondori-Teranouchisagaru, Kamigyo-Ku, Kyoto, Japan; 2 Department of Medical Statistics, Research & Development Center, Osaka Medical and Pharmaceutical University, 2-7 Daigaku-Machi, Takatsuki, Osaka Japan; 3 Department of Social Epidemiology, Graduate School of Medicine and School of Public Health, Kyoto University, Yoshida-Konoe-Cho, Sakyo-Ku, Kyoto, Kyoto Japan

**Keywords:** Low-income, Poverty, Health Related Quality of Life, Free/Low-Cost Medical Care program, Public Assistance

## Abstract

**Background:**

Low-income is one of the well-established determinants of people’s health and health-related behavior, including susceptibility to the coronavirus disease 2019 (COVID-19) infection. Two social welfare services are available in Japan to support financial and medical care among low-income patients: Public Assistance (PA), which provide both minimum income and medical costs; and Free/Low-Cost Medical Care (FLCMC), wherein only medical costs were covered. In this study, changes in Health-Related Quality of Life (HRQOL) scores of low-income patients on PA and FLCMC, before and after COVID-19 pandemic, were described and compared against those that are not utilizing the said services (comparison group) to evaluate the contribution of social welfare services in protecting the HRQOL of the beneficiaries during the pandemic.

**Methods:**

We used repeated cross-sectional data of adult beneficiaries of FLCMC and PA, as well as those without social welfare services, who regularly visit the Kamigyo clinic in Kyoto, Japan. We collected the data from 2018 and 2021 using a questionnaire on patients’ socioeconomic attributes and the Japanese version of Medical Outcomes Study 12-Item Short Form Health Survey (SF-12). The Japanese version of SF-12 can calculate the three components scores: physical health component summary (PCS), the mental health component summary (MCS), and the role-social component summary (RCS), which can be transformed to a 0–100 range scale with a mean of 50 and standard deviation of 10.

**Results:**

Data of 200 and 174 beneficiaries in 2018 and 2021, respectively, were analyzed. Low-income patients on social welfare services had lower PCS, and RCS than the comparison group in both years. Multiple linear regression analyses with cluster-adjusted standard error estimator showed that the decline in MCS was significantly higher among FLCMC beneficiaries than in those without welfare services (Beta: -4.71, 95% Confidence Interval [CI]: –5.79 to -3.63, *p* < 0.01), and a decline in MCS among PA recipients was also observed (Beta: -4.27, 95% CI: -6.67 to -1.87 *p* = 0.02).

**Conclusions:**

Low-income beneficiaries of social welfare may have experienced mental health deterioration during the COVID-19 pandemic. To maintain healthy lives during the pandemic, additional support on mental health for low-income recipients of social welfare services may be required.

**Supplementary Information:**

The online version contains supplementary material available at 10.1186/s12889-022-14597-5.

## Background

Socioeconomic status is one of the well-established determinants of a person’s health and health behavior, which considerably affect quality of life (QOL) [1]. In particular, individuals with low-income face challenges in maintaining healthy lives as they tended to have a higher risk of health-related problems [1], difficulties in health care access [2], unhealthy behaviors such as smoking and infrequent exercise [3], lower cancer screening rates [4], and higher vaccine hesitancy rates [5]. Furthermore, they typically suffer from multidimensional difficulties of poverty-related issues, including social isolation and time poverty [6–8]. Low-income individuals are susceptible to the spread of various infections, including coronavirus disease 2019 (COVID-19) [9, 10]. COVID-19 has been reported to reduce physical activity, worsen mental function, and escalate states of social isolation especially among low-income people, which in turn has deteriorated their QOL further [11, 12].

As low-income is one of the strong determinants of health, governments in developed countries have several welfare programs that can provide financial support to improve livelihoods and healthcare access among the impoverished populations. In Japan, there are two well-known welfare services called Public Assistance (PA; ‘seikatsu-hogo’ in Japanese), and Free/Low-Cost Medical Care (FLCMC; ‘muryo-teigaku-shinryo’ in Japanese). PA is a governmental program for people who are living below the poverty line and without any assets. Approximately 1.7% of the Japanese population is enrolled in the PA program [13]. To evaluate the eligibility for PA, a rigorous test for means of each potential household is conducted by the local municipal welfare office (i.e., whether they are living below the poverty line, their ability to work, the financial support they receive from relatives, and their use of any other welfare services). Meanwhile, the FLCMC program is a voluntary program governed by the Social Welfare Act, provided by designated healthcare institutions. The purpose of FLCMC included providing free or low-cost medical care to people with financial difficulties so that they are not restricted in their access to necessary medical care due to financial reasons [14]. FLCMC applicants are screened individually by their corresponding institution, and eligible recipients are exempted from out-of-pocket medical payment at the designated institutions, which cover their medical care costs. Institutions can earn the benefits of tax exemptions depending on the proportion of patients who use their FLCMC program. Thus, we can regard PA recipients as impoverished people benefited with minimum income protection and livelihood including medical care costs, while FLCMC recipients as financially restricted individuals who are benefited only with medical care costs.

Previous global studies have shown that recipients of PA have more unfavorable health conditions when compared to general populations [15]. For example, PA recipients are more likely to have diabetes [16], depressive symptoms [17], and suicidal ideation/behavior [18]. Similarly, recipients of FLCMC in Japan are more likely to have lower health-related quality of life (HRQOL) [19–21]. These previous studies analyzed and reported pre-COVID-19 pandemic data. Although the health impacts of the pandemic have been remarkable among low-income individuals [9, 22], there are no studies investigating impact of welfare services on these populations, who might receive protective health effects.

Therefore, the purpose of this study was to compare the changes in HRQOL during the COVID-19 pandemic among low-income patients on PA and FLCMC, as well as those not benefiting from social welfare services, through repeated cross-sectional surveys in a single center in Kyoto, Japan.

## Methods

### Design of the study

We used repeated cross-sectional data obtained from adult beneficiaries of FLCMC and PA programs as well as those not utilizing social welfare services, who used the Kamigyo clinic in Kyoto, Japan in FY 2017 and 2020. Kamigyo Clinic is situated in Kamigyo-ward, which is a historically prosperous administrative center of Kyoto prefecture, where the capital was established anciently. Kamigyo-ward has a total population of 85,000; of which, 1.6% (approximately 1500 people) are receiving public assistance. The aging population rate is 26.1%.

### Participants

All beneficiaries of FLCMC and PA who visited Kamigyo Clinic in 2017 and 2020 was included in this study. In FY 2017, there were 226 and 185 on FLCMC and PA, respectively. Meanwhile, in FY 2020, there were 253 and 161 patient on FLCMC and PA, respectively. For a comparison of the recipients of FLCMC and PA, we sampled patients in Kamigyo Clinic who were not receiving social welfare programs (comparison group). Kamigyo Clinic has a list of patients not utilizing social welfare services, which include 1637 and 1742 patients in FY 2017 and 2020, respectively. To maintain representativeness of comparison group, patients not on social welfare services were randomly sampled from the outpatients list of each year by using the random function of Microsoft Excel for Mac (ver.16.54). We sampled the patients without social welfare services as much as recipients of FLCMC. We collected the repeated data at the two time points of 2018 (before COVID-19), and 2021 (during COVID-19).

### Data Collection

**Fig. 1:**
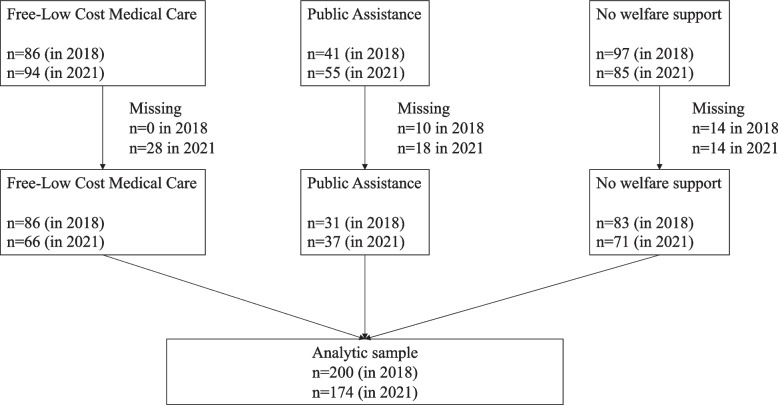
Flow chart of study participants

We conducted a self-questionnaire survey on patients’ social background, including working status and household number as well as the HRQOL using the Japanese version of Medical Outcomes Study 12-Item Short Form Health Survey (SF-12) [23]. Since this repeated cross-sectional survey was obtained through an anonymous questionnaire, we could not reconcile the 2017 and 2020 data and medical records of patients in Kamigyo Clinic. The survey was completed in the Japanese language and translated in English during the analyses. The survey was conducted through mail. A reply envelope was enclosed, and the postage was borne by the facility. Although imputation method in missing data of the SF-12 questionnaire has been discussed [24], the Japanese version of SF-12 recommends not to impute the missing value [25]. A number of FLCMC or PA beneficiaries have remarkable missing information, and thus we excluded participants who have missing data in the variables used in this study (Fig. 1).

### Variables

#### Outcome variables

We used the HRQOL scores to determine the outcomes. Briefly, we used the SF-12 questionnaire that contains a combination of positively- (higher scores indicate better health) and negatively worded response scales [25, 26]. The scale scores are calculated by the responses across scale items and transformed into a 0–100 scale by computerized scoring algorithms (with a mean of 50 and SD of 10). The Japanese version of SF-12 calculates the scores of the three components: the physical health component summary (PCS), the mental health component summary (MCS), and the role-social component summary (RCS). In the original English version of the SF-12, two component summary scores (PCS and MCS) can be calculated from the eight subscales. However, the use of this two-component summary score was not recommended in Asian countries, including Japan due to the differences in factor structures. A three-component scoring method was developed by adding role/social dimensions (RCS) to PCS and MCS, based on a large-scale population study in Japan [26]. In this study, these components were measured using the scoring method [23].

#### Explanatory variables

We used the variable of availing status of welfare services (FLCMC, PA, and no welfare services) and the year (2018/2021).

#### Covariates

We used sex (male/female), age (continuous), household composition (living alone or not), and working status (working or not) as covariates.

### Statistical analysis

First, we described the characteristics and responses to SF-12 questionnaire of the study participants in two-time survey across the availing statuses of social welfare services. Second, we performed univariable linear regression analysis, and multiple linear regression analysis adjusting for the covariates to calculate crude and multivariable-adjusted Beta estimates of the changes in HRQOL scores and the 95% confidence interval (CI) of explanatory variables. In addition, we included an interaction term of availing status of social welfare services and year variable in multiple regression analyses to investigate the heterogeneity of changes in HRQOL scores by welfare service recipients before and after the COVID-19 pandemic and illustrated the results in the figure. We used cluster-adjusted standard error estimator in all regression analyses to control the influence of clustered data derived from the study participants in single-center nested in the survey year to prevent potential alpha errors due to narrowing CI by clustered data. All analyses were performed using STATA MP Ver.17 (Stata Corp., College Station, TX, USA).

### Ethical considerations

All study participants provided informed consent. This study protocol was approved by the Ethics Committee of the Kyoto Min-iren Chuo Hospital (Approval No: 124).

## Results

From the 2018 data, 226, 185, and 226 patients on FLCMC, PA, and without welfare services, respectively, were included in the study. Meanwhile, from the 2021 data, 253, 161, and 300 patients on FLCMC, PA, and without welfare services, respectively, were included. The response rates were 42.9% in the FLCMC group, 22.2% in the PA group and 38.1% in the no welfare services group in 2018, and 33.6% in the FLCMC group, 34.2% in the PA group and 31.3% in the no welfare services group in 2021.

Among the participants in 2018, a total of 200 patients’ data (excluding missing) were obtained. Among them, 86 (43.0%) patients were on FLCMC, and 31(15.5%) patients were on PA. Among the participants in 2021, a total of 174 patients’ data (excluding missing) were obtained. Among them, 83 (37.9%) patients were on FLCMC, 37 (21.3%) patients were on PA. The mean scores of PCS and RCS were lower among beneficiaries of FLCMC and PA than those without welfare services in both 2018 and 2021 (Table 1). The mean scores of MCS were lower among beneficiaries of FLCMC and PA than patients without welfare services in 2021 (Table 1), particularly in general health (GH) and vitality (VT) domains of the SF-12. (Supplementary Table 1).

**Table 1:** Baseline characteristics of study participants availing welfare services by year

	2018 (*n* = 200)			2021(*n* = 174)		
	FLCMC (*n* = 86)	PA (*n* = 31)	No welfare services (*n* = 83)	FLCMC (*n* = 66)	PA (*n* = 37)	No welfare services (*n* = 71)
HRQOL scores (Mean, SD)						
PCS	36.5, 14.5	34.3, 15.6	42.4, 12.8	36.5, 17.5	36.4, 12.7	44.3, 13.6
MCS	51.8, 12.0	51.0, 12.4	52.0, 8.3	48.8, 8.3	46.6, 9.5	51.1, 8.6
RCS	43.1, 13.6	39.3, 13.2	46.3, 11.8	41.7, 13.9	37.7, 13.5	44.1, 10.9
Age (Median [IQR])	76 [69–82]	72 [65–78]	74 [67–83]	78 [71–83]	70 [66–77]	72 [63–79]
Sex						
Male	44	16	36	32	22	32
Female	42	15	47	34	15	39
Household composition						
Living alone	33	22	23	23	32	16
Not living alone	53	9	60	43	5	55
Working status						
Working	24	4	33	19	3	29
Not working	62	27	50	47	34	42

*SD* standard deviation, *IQR* Interquartile Range, *FLCMC* Free/ Low-Cost Medical Care Program, *PA* Public Assistance, *PCS* Physical Component Summary, *MCS* Mental Component Summary, *RCS* Role-social Component Summary

The univariable linear regression analysis showed that use of social welfare services (both PA and FLCMC) were inversely associated with PCS, MCS and RCS scores (Table 2). In year 2021, MCS and RCS scores were lower when compared against that of 2018 (Table 2). The results of multiple linear regression showed that adjusted Beta estimates of PCS, MCS, and RCS were lower in FLCMC recipients than in patients without welfare services (PCS -5.55, 95% CI -7.20 to -3.91, *p* = 0.01; MCS -2.61, 95% CI -4.24 to -0.98, *p* = 0.02; RCS -2.55, 95% CI -3.28 to -1.83, *p* < 0.01). Moreover, among the PA beneficiaries, scores in PCS, MCS and RCS were lower than in patients without welfare services (PCS -7.33, 95% CI -10.26 to -4.39, *p* = 0.01; MCS -2.88, 95% CI -6.74 to 0.97, *p* = 0.08; RCS -7.63, 95% CI -11.86 to -3.41, *p* = 0.02) (Table 2).

**Table 2:** Results of univariable and multivariable regression analysis on HRQOL scores of study participants

	PCS				MCS				RCS			
	Beta	95%CI		*P*	Beta	95%CI		*P*	Beta	95%CI		*P*
Univariable Regression												
* Explanatory Variable*												
Use of welfare support (Ref: not using)												
FLCMC	-6.75	-10.01	-3.50	< 0.01*	-2.00	-4.24	0.24	0.08	-2.80	-5.67	0.06	0.06
PA	-7.80	-11.94	-3.66	< 0.01*	-2.99	-5.83	-0.14	0.04*	-6.89	-10.53	-3.24	< 0.01*
Year (Ref: 2018)												
2021	1.05	-1.36	3.46	0.20	-3.22	-9.55	3.12	0.16	-2.04	-3.71	-0.37	0.04*
* Covariates*												
Male (Ref: female)	0.86	-5.01	6.73	0.59	-1.38	-8.55	5.80	0.50	-1.35	-6.06	3.35	0.34
Age (continuous)	-0.37	-0.64	-0.10	0.03*	0.16	-0.12	0.44	0.14	-0.17	-0.42	0.09	0.11
Working (Ref: not working)	-6.97	-14.68	0.73	1.08	-1.47	3.64	0.21	0.31	-5.78	-13.29	1.73	0.08
Living alone (Ref: not living alone)	3.68	-1.39	8.75	0.09	0.27	-7.62	8.16	0.90	0.44	-7.37	8.26	0.83
Multivariable Regressions												
* Explanatory Variable*												
Use of welfare support (Ref: not using)												
FLCMC	-5.55	-7.20	-3.91	0.01*	-2.61	-4.24	-0.98	0.02*	-2.55	-3.28	-1.83	< 0.01*
PA	-7.33	-10.26	-4.39	0.01*	-2.88	-6.74	0.97	0.08	-7.63	-11.86	-3.41	0.02*
Year (Ref: 2018)												
2021	0.97	-1.34	3.29	0.21	-3.09	-10.38	4.20	0.21	-1.87	-3.74	0.01	0.05
* Covariates*												
Male (Ref: female)	0.92	-5.30	7.13	0.59	-1.83	-8.67	5.00	0.37	-1.71	-6.29	2.86	0.25
Age (continuous)	-0.34	-0.52	-0.16	0.02*	0.16	-0.18	0.50	0.18	-0.13	-0.36	0.09	0.13
Working (Ref: not working)	-1.78	-11.90	8.35	0.53	0.03	-3.19	3.25	0.97	-3.36	-10.66	3.94	0.19
Living alone (Ref: not living alone)	1.16	-2.70	5.02	0.33	-0.49	-7.85	6.87	0.80	-2.63	-9.03	3.77	0.22

*Beta* Beta estimate, *FLCMC* Free/ Low Cost Medical Care Program, *PA* Public Assistance, *PCS* Physical Component Summary, *MCS* Mental Component Summary, *RCS* Role-social Component Summary *P<0.05

The multiple regression with an interaction model (use of welfare services and year) showed that the decline in MCS scores in 2021 was significantly higher among FLCMC recipients (-4.71, 95% CI -5.79 to -3.63, *p* < 0.01) when compared with patients without welfare services. Similarly, the decline in MCS scores in 2021 among PA beneficiaries was larger when compared with patients without welfare services (-4.27, 95% CI -6.67 to -1.87, *p* = 0.02) (Table 3, Supplementary Fig. 1).

**Table 3:** Results of multivariable regression analysis including statistical interaction term on HRQOL scores of study participants

	PCS				MCS				RCS			
	Beta	95%CI		*P*	Beta	95%CI		*P*	Beta	95%CI		*P*
Multivariable Regression												
* Explanatory Variable*												
Use of welfare support (Ref: not using)												
FLCMC	-5.27	-6.80	-3.74	0.01*	-0.51	-1.56	0.53	0.17	-3.12	-4.22	-2.03	0.01*
PA	-8.24	-10.43	-6.05	< 0.01*	-0.79	-3.78	2.21	0.38	-7.87	-11.88	-3.85	0.01*
Year (Ref: 2018)												
2021	0.90	0.36	1.44	0.02*	-0.40	-1.38	0.57	0.22	-2.47	-3.12	-1.82	< 0.01*
Use of welfare support (Ref: not using) x Year (Ref: 2018)												
FLCMCx2021	-0.64	-1.13	-0.14	0.03*	-4.71	-5.79	-3.63	< 0.01*	1.27	0.24	2.30	0.03*
PAx2021	1.80	0.22	3.38	0.04*	-4.27	-6.67	-1.87	0.02*	0.50	-0.44	1.43	0.15
* Covariates*												
Male (Ref: female)	0.96	-5.32	7.24	0.58	-1.83	-8.61	4.96	0.37	-1.73	-6.36	2.90	0.25
Age (continuous)	-0.34	-0.52	-0.16	0.01*	0.17	-0.16	0.50	0.16	-0.14	-0.36	0.09	0.13
Working (Ref: not working)	-1.80	-12.04	8.43	0.53	-0.05	-3.08	2.99	0.95	-3.34	-10.70	4.03	0.19
Living alone (Ref: not living alone)	1.24	-2.31	4.80	0.27	-0.57	-7.56	6.41	0.76	-2.64	-8.99	3.72	0.22

*Beta* Beta estimate, *FLCMC* Free/ Low Cost Medical Care Program, *PA* Public Assistance, *PCS* Physical Component Summary, *MCS* Mental Component Summary, *RCS* Role-social Component Summary

^*^P < 0.05

## Discussion

Our study found that low-income beneficiaries of FLCMC and PA had lower PCS and RCS scores than comparison group, both before and after the pandemic. Additionally, MCS scores in 2021 among FLCMC and PA recipients have declined predating the pandemic as compared with the comparison group. The strength of this study was that by using baseline data in 2018, we can evaluate the changes of health status before and after the COVID-19 pandemic among the low-income patients on social welfare services.

### Findings in context

Our results indicating lower PCS scores among PA and FLCMC beneficiaries were consistent with previous studies, which were reported before COVID-19 pandemic [19–21]. Recent studies revealed that PA beneficiaries have a higher prevalence of chronic health conditions, such as diabetes and mental illness [16–18]. Although we could not identify the diseases of the participants in this study, our study added the knowledge that existing evidence can also be applied to low-income social welfare recipients during COVID-19 pandemic. The findings that the RCS scores were lower among both PA and FLCMC recipients may be due to social isolation among these population. Previous literatures have shown that both PA and FLCMC beneficiaries tend to live alone and to have fewer opportunities of interpersonal exchange [19, 20, 27]. The psychological impact of COVID-19 pandemic to socially vulnerable populations may probably explain the decline in MCS scores among FLCMC and PA beneficiaries [28, 29]. The social welfare services which financially support low-income patients might not sufficiently mitigate their psychological stress [30, 31].

### Practice and policy implications

Our study provided novel evidence that the score of MCS declined among FLCMC and PA beneficiaries. Mental health deterioration among low-income individuals during the COVID-19 pandemic was reported globally [32–34]. Our study demonstrated that social welfare services, which financially or medically assist recipients, might be insufficient to mitigate the impact of communicable diseases on the mental health of low-income beneficiaries. Organizations involved in the support for these population, such as medical institutions and welfare offices should consider interventions that can maintain their mental health. For example, it has been reported that community-based activities providing simultaneous medical and social care can improve mental health and well-being among socially vulnerable populations [35, 36]. The findings of significant decline of MCS scores among FLCMC and PA recipients suggested that social care, including financial support for their livelihood might be important in improving mental health for impoverished population [31].

### Limitations

Our study had several limitations. First, the response rates among each population were low (42.5% in total) and we conducted complete case analyses; the findings in this study may be biased. Imputation method was considered; however, due to a large proportion of missing data among FLCMC and PA beneficiaries in 2021, as well as the recommendation in the Japanese version of SF-12 [25], we did not impute the missing data. A lower response rate and a higher missing proportion was observed in FLCMC and PA users. Moreover, beneficiaries who responded to the survey can be physically and mentally health, and thus the findings in the study may have a bias toward underestimation.

Second, because the study design was repeated cross-sectional, the participants investigated in 2018 and 2021 were not always consistent. We could not argue that FLCMC and PA beneficiaries experienced mental health deterioration. Unmeasured confounders between utilization of social welfare services and mental health deterioration may exist. For example, low-income individuals might be in states of social isolation, which can affect their mental health. Although we included the variables associated with social isolation (i.e., living alone: exclusion from the community of the family; working or not: exclusion from the community of the workplace) and RCS scores, decline in MCS scores among recipients still existed (Supplementary Table 2). Possible confounder was resilience to mental health problems or coping skill to psychological stress [37, 38] associated with educational attainment, which we could not obtain in this survey. Further, there may be a potential mechanism of reverse causation that people who are not beneficiaries of FLCMC or PA in 2018 may suffer from mental health deterioration, and thus are receipts of social welfare support services in 2021.

Third, the data used in this study was obtained through anonymous surveys. Thus, we could not reconcile the medical records of the patients in Kamigyo clinic. Consequently, only the HRQOL scores were considered. Comparability between three groups was not ensured. This point should be improved to consider health conditions of the study participants in future research.

Fourth, the income level of comparison group was not considered. Compared with FLCMC and PA groups, patients in comparison group have a higher income. This assumption is based on a rationale that if their income was low, the patients would be included in either FLCMC or PA group in Kamigyo Clinic. A higher income equates to preferable health conditions, and this may over-estimate the effect of FLCMC and PA. Future study recruiting similar income patients but not on FLCMC or PA across different institution will be required.

Finally, generalizing the data obtained from this study is limited because this study used data from a single healthcare institution in Kyoto.

## Conclusion

Low-income patients on social welfare services (FLCMC and PA) had lower PCS and RCS scores and experienced decline in MCS scores during the COVID-19 pandemic than in those not utilizing social welfare services. Among low-income patients, social welfare services may not sufficiently protect the mental health of the beneficiaries from communicable diseases pandemic. Further strategy supporting mental health among low-income population may be required.

## Supplementary Information

**Additional file 1: Supplementary Table 1.**Summary of the scores for each domain of the Japanese versions of SF-12. **Supplementary Figure 1.** Prediction margins of HRQOL scores availing statuses on social welfare services and before-after COVID-19. **Supplementary Table 2**. Results of multivariable regression analysis adjusted by PCS and RCS on MCS scores of study participants.

## Data Availability

The data used in this study were collected from patients of Kamigyo Clinic which are not publicly available. The data are available from the authors upon reasonable request, with the permission of the Kamigyo Clinic. Please contact to the corresponding author for the request.

## Abbreviations

COVID-19: Coronavirus disease 2019
CI: Confidence interval
FLCMC: Free or Low-Cost Medical Care
PA: Public Assistance
PCS: Physical Component Summary
MCS: Mental Component Summary
RCS: Role-social Component Summary
QOL: Quality of life
HRQOL: Health related quality of life
