# Supplementary material for: Changes in health-related quality of life scores among low-income patients on social welfare programs in Japan during the COVID-19 pandemic: a single-center repeated cross-sectional study
Source: BMC Public Health. 2022 Nov 22;22:2147. doi: 10.1186/s12889-022-14597-5 (PMC9682633; doi:10.1186/s12889-022-14597-5)
Supplement: Supplementary file 1 — Additional file 1: Supplementary Table 1.Summary of the scores for each domain of the Japanese versions of SF-12. Supplementary Figure 1. Prediction margins of HRQOL scores availing statuses on social welfare services and before-after COVID-19. Supplementary Table 2. Results of multivariable regression analysis adjusted by PCS and RCS on MCS scores of study participants. [file 12889_2022_14597_MOESM1_ESM.docx]

| **Supplementary Table 1.** Summary of the scores for each domain of the Japanese versions of SF-12 | | | | | | | |
| --- | --- | --- | --- | --- | --- | --- | --- |
|  | 2018 (N=200) |  |  |  | 2021(N=174) |  |  |
|  | FLCMC | PA | No welfare support |  | FLCMC | PA | No welfare support |
|  | (n=86) | (n=31) | (n=83) |  | (n=66) | (n=37) | (n=71) |
| HRQOL scores (Mean, SD) |  |  |  |  |  |  |  |
| PCS | 36.5, 14.5 | 34.3, 15.6 | 42.4, 12.8 |  | 36.5, 17.5 | 36.4, 12.7 | 44.3, 13.6 |
| MCS | 51.8, 12.0 | 51.0, 12.4 | 52.0, 8.3 |  | 48.8, 8.3 | 46.6, 9.5 | 51.1, 8.6 |
| RCS | 43.1, 13.6 | 39.3, 13.2 | 46.3, 11.8 |  | 41.7, 13.9 | 37.7, 13.5 | 44.1, 10.9 |
| Scores in each domain of SF-12  (Mean, SD) |  |  |  |  |  |  |  |
| Physical functioning (PF) | 38.8, 16.4 | 35.6, 16.0 | 45.1, 13.4 |  | 36.0, 16.7 | 36.5, 12.9 | 45.2, 13.7 |
| Role Physical (RP) | 39.2, 14.3 | 33.5, 14.3 | 41.9, 13.4 |  | 35.6, 14.5 | 33.6, 13.8 | 42.7, 12.2 |
| Bodily Pain (BP) | 38.1, 13.7 | 36.2, 12.8 | 42.9, 13.7 |  | 37.4, 14.2 | 35.5, 12.8 | 43.9, 13.1 |
| General Health (GH) | 42.5, 10.1 | 42.6, 11.8 | 45.1, 9.3 |  | 40.4, 9.7 | 39.0, 9.0 | 46.8, 9.2 |
| Vitality (VT) | 50.8, 12.7 | 47.3, 11.6 | 49.4, 10.7 |  | 42.2, 9.9 | 42.8, 10.7 | 48.9, 11.4 |
| Social Functioning (SF) | 45.8, 11.5 | 40.4, 15.0 | 47.4, 11.2 |  | 40.2, 14.1 | 39.3, 13.9 | 44.0, 10.9 |
| Role Emotional (RE) | 38.1, 13.0 | 38.2, 14.6 | 43.3, 12.6 |  | 38.6, 14.1 | 33.4, 13.0 | 44.5, 12.1 |
| Mental Health (MH) | 47.5, 8.8 | 43.3, 13.7 | 51.4, 8.8 |  | 44.4, 11.3 | 42.6, 12.4 | 50.3, 9.8 |
| SD standard deviation, FLCMC Free/ Low Cost Medical Care Program, PA Public Assistance, PCS Physical Component Summary, MCS Mental Component Summary, RCS Role-social Component Summary. | | | | | | | |

**Supplementary Figure 1.** Prediction margins of HRQOL scores availing statuses on social welfare services and before-after COVID-19


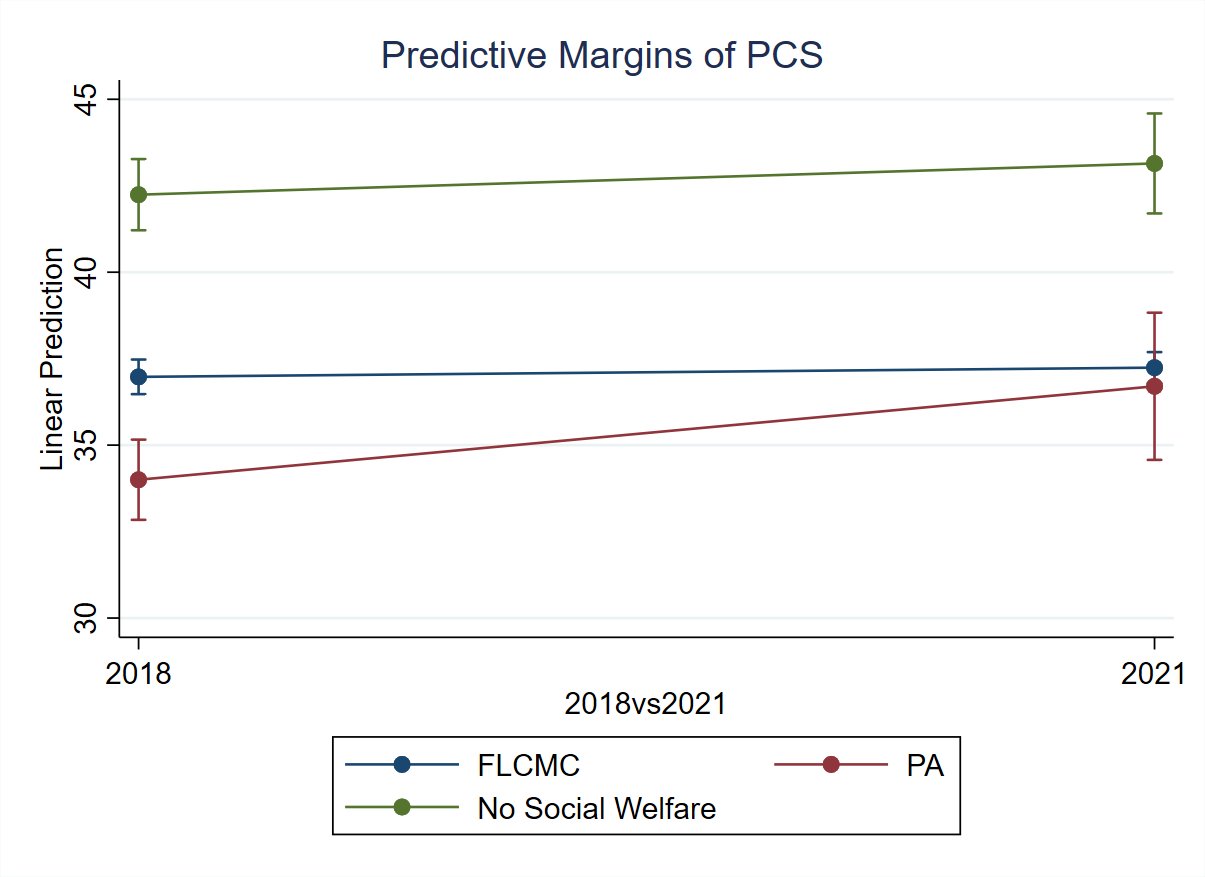
 **
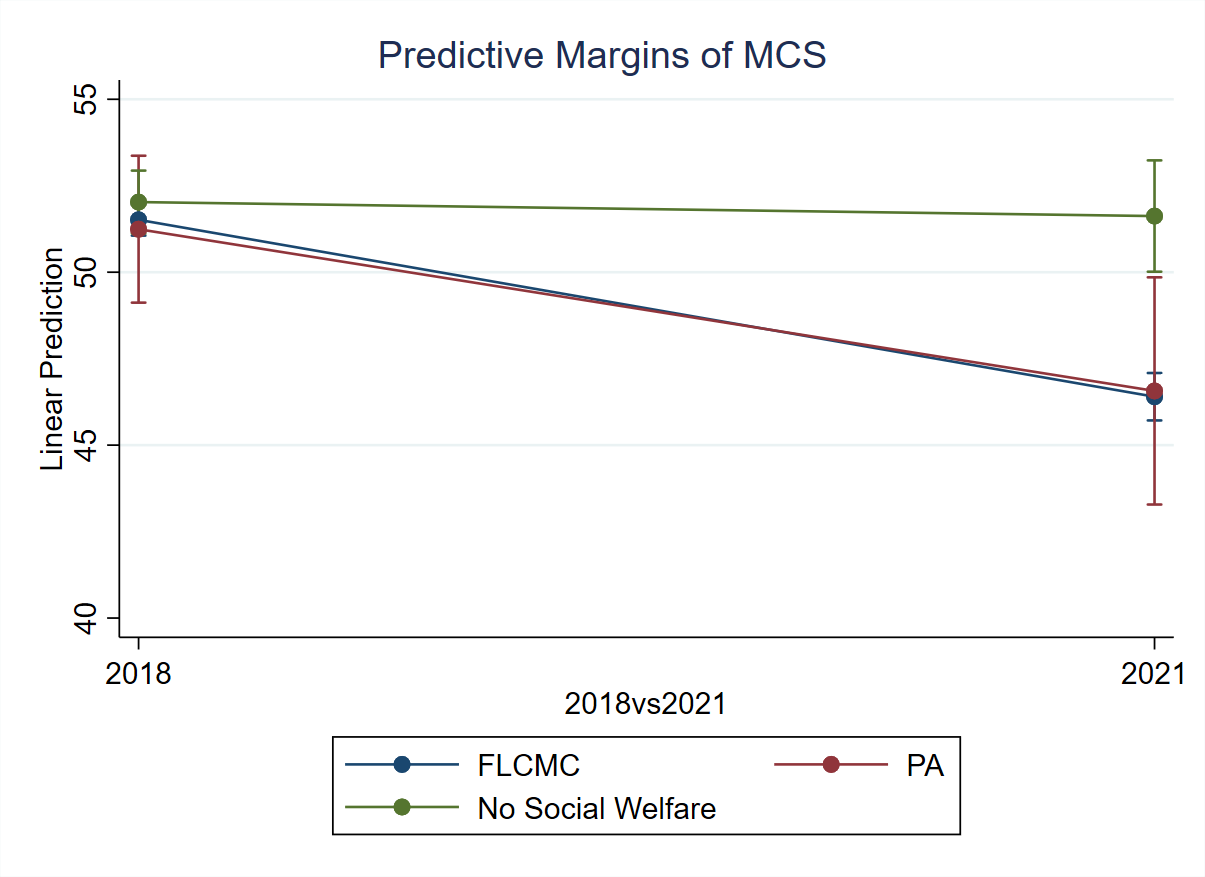

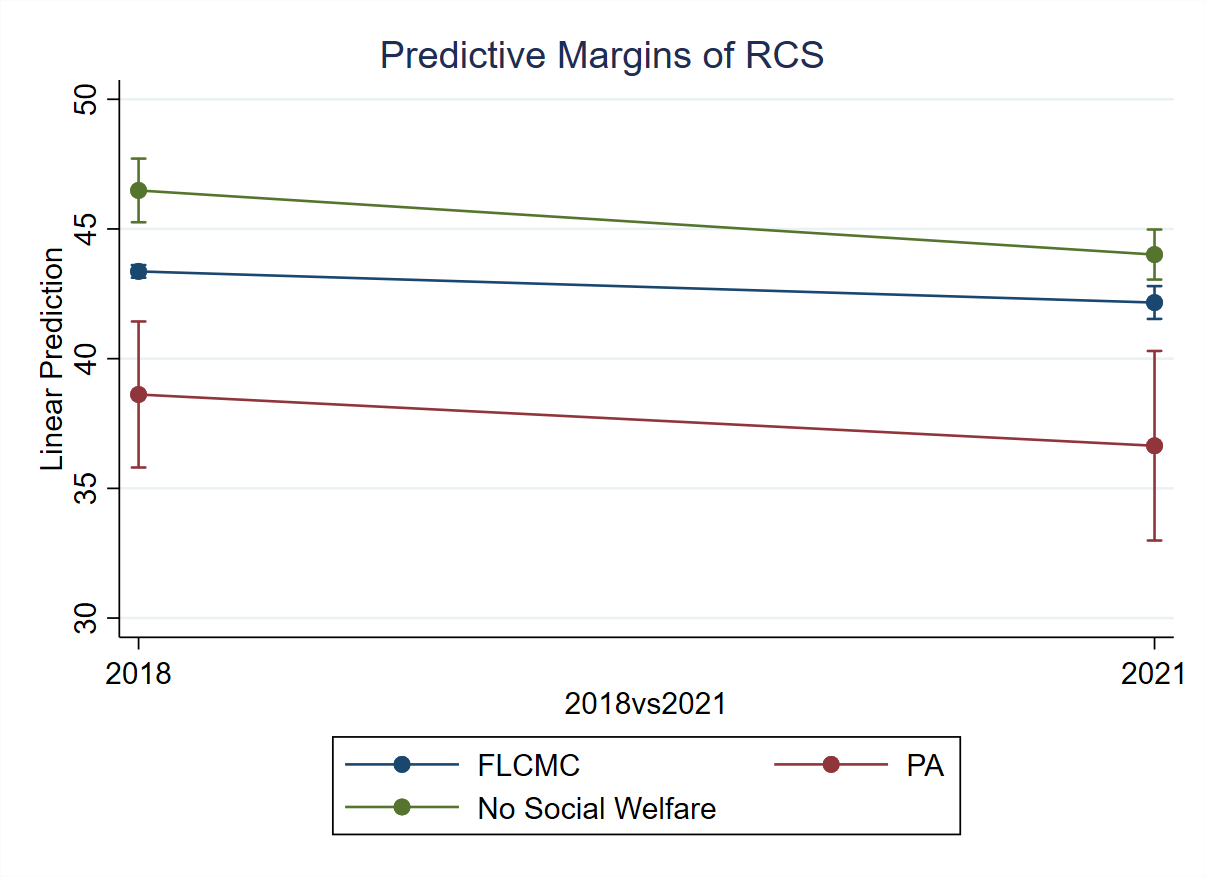
**

| **Supplementary Table 2**. Results of multivariable regression analysis adjusted by PCS and RCS on MCS scores of study participants. | | | | |
| --- | --- | --- | --- | --- |
|  | MCS |  |  |  |
|  | Beta | 95%CI |  | p-value |
| Multivariable Regression |  |  |  |  |
| *Explanatory Variable* |  |  |  |  |
| Use of welfare support (Ref: not using) |  |  |  |  |
| FLCMC | -1.57 | -4.90 | 1.75 | 0.18 |
| PA | -2.62 | -10.31 | 5.07 | 0.28 |
| Year (Ref: 2018) |  |  |  |  |
| 2021 | -0.40 | -1.47 | 0.66 | 0.24 |
| Use of welfare support (Ref: not using) x Year (Ref: 2018) |  |  |  |  |
| FLCMCx2021 | -4.74 | -5.82 | -3.66 | <0.01* |
| PAx2021 | -3.94 | -5.50 | -2.38 | 0.01* |
| *Covariates* |  |  |  |  |
| PCS | -0.17 | -0.35 | 0.01 | 0.06 |
| RCS | -0.06 | -0.64 | 0.52 | 0.70 |
| Male (Ref: female) | -1.77 | -8.43 | 4.89 | 0.37 |
| Age (continuous) | 0.10 | -0.15 | 0.35 | 0.22 |
| Working (Ref: not working) | -0.55 | -6.84 | 5.74 | 0.74 |
| Living alone (Ref: not living alone) | -0.52 | -8.31 | 7.26 | 0.80 |
| Beta: Beta estimate, FLCMC Free/ Low Cost Medical Care Program, PA Public Assistance, PCS Physical Component Summary, MCS Mental Component Summary, RCS Role-social Component Summary. * p<0.05 | | | | |
